# Role of advanced glycation end products in the longitudinal association between muscular strength and psychotic symptoms among adolescents

**DOI:** 10.1038/s41537-022-00249-5

**Published:** 2022-04-27

**Authors:** Kazuhiro Suzuki, Syudo Yamasaki, Mitsuhiro Miyashita, Shuntaro Ando, Kazuya Toriumi, Akane Yoshikawa, Miharu Nakanishi, Yuko Morimoto, Sho Kanata, Shinya Fujikawa, Kaori Endo, Shinsuke Koike, Satoshi Usami, Masanari Itokawa, Shinsuke Washizuka, Mariko Hiraiwa-Hasegawa, Herbert Y. Meltzer, Kiyoto Kasai, Atsushi Nishida, Makoto Arai

**Affiliations:** 1 Schizophrenia Research Project, Department of Psychiatry and Behavioral Sciences, Tokyo Metropolitan Institute of Medical Science, Tokyo, Japan; 2 Department of Psychiatry, Shinshu University School of Medicine, Matsumoto, Japan; 3 Department of Psychiatry, Tokyo Metropolitan Matsuzawa Hospital, Tokyo, Japan; 4 Department of Community Mental Health, Shinshu University School of Medicine, Matsumoto, Japan; 5 Research Center for Social Science & Medicine, Tokyo Metropolitan Institute of Medical Science, Tokyo, Japan; 6 Department of Neuropsychiatry, Graduate School of Medicine, The University of Tokyo, Tokyo, Japan; 7 University of Tokyo Institute for Diversity and Adaptation of Human Mind (UTIDAHM), University of Tokyo, Tokyo, Japan; 8 Center for Research and Development on Transition from Secondary to Higher Education, The University of Tokyo, Tokyo, Japan; 9 Department of Evolutionary Studies of Biosystems, The Graduate University for the Advanced Studies, SOKENDAI, Hayama, Japan; 10 Department of Psychiatry and Behavioral Sciences, Northwestern Feinberg School of Medicine, Chicago, IL USA; 11 The International Research Center for Neurointelligence (WPI-IRCN) at The University of Tokyo Institutes for Advanced Study (UTIAS), University of Tokyo, Tokyo, Japan

**Keywords:** Psychosis, Biomarkers, Developmental biology

## Abstract

Muscular strength, assessed by handgrip, is a risk indicator for psychiatric disorders, including psychosis. However, the biological mechanisms underlying this association remain unclear. Since advanced glycation end products (AGEs) play a key role in skeletal muscle underdevelopment and psychosis, we examined the role of AGEs in the longitudinal association between muscular strength and psychotic symptoms among adolescents. We first evaluated the direction of the relationship between handgrip strength and urine levels of pentosidine, a representative AGEs in a population-based birth cohort of 1,542 adolescents at ages 12 and 14. Then, we examined the role of AGEs in the longitudinal association between handgrip strength and thought problems (TP), as a psychotic symptom indicator, in a subsample of 256 adolescents at ages 13 and 14. An autoregressive cross-lagged model revealed that handgrip strength at age 12 negatively predicted pentosidine levels at age 14 (β = −0.20, *p* < 0.001), whereas pentosidine levels at age 12 did not predict handgrip strength at age 14 (β = 0.04, *p* = 0.062). Moreover, pentosidine levels had a significant indirect effect on the relationship between handgrip strength and TP (standard indirect effect = −0.051, *p* = 0.012), which remained significant after adjusting for gender and preceded TP and pentosidine levels. Thus, adolescents with low muscular strength are at a high risk of developing psychotic symptoms, which could be mediated by AGEs. Future studies need to investigate whether interventions focused on muscular strength prevent the accumulation of AGEs and thereby prevent the development of psychosis.

## Introduction

Patients with schizophrenia have been consistently shown to present a higher incidence of muscular system underdevelopment^1^. Many studies have confirmed Kretchmer’s observation of a greater incidence of asthenic or leptosomatic constitutions, which are characterized by narrow muscles, small shoulders, a flat chest, and little muscular development, in schizophrenic or pre-schizophrenic individuals^1^. These early observations have been supported by later studies using muscle biopsy and/or electromyography, which showed abnormalities in both muscle function and muscle morphology in schizophrenia^2–7^. These skeletal muscle abnormalities include evidence of neurodevelopmental and neurodegenerative processes due to abnormalities in the brain that may be related to core symptoms of schizophrenia, such as thought disorder^8^.

Handgrip strength, determined using a handgrip dynamometer, is a simple and accurate measure of muscular strength, especially in children and adolescents^9,10^. A large cohort study of more than a million Swedish adolescents reported that lower muscular strength in adolescents, which was assessed by handgrip, is a risk factor for later psychiatric diagnosis, including schizophrenia^11^. However, the biological mechanisms underlying the association between muscular strength and psychosis remain unclear.

Advanced glycation end products (AGEs) are derived from irreversible non-enzymatic modification of proteins and amino acids with reducing sugars. Accumulation of AGEs causes various diabetic complications, including neuropathy and Alzheimer’s disease, via cytotoxicity by glycation and oxidation stress^12^. AGEs have been suggested to be responsible for the association between muscle strength and psychotic phenomena. Previous cross-sectional studies have reported an association between AGE accumulation and weak muscle strength^13,14^. Lower muscle strength increases AGE accumulation due to exacerbated insulin resistance, given that skeletal muscle tissue is the predominant site of insulin-mediated glucose uptake^15^. Furthermore, studies have reported that patients with schizophrenia have higher AGE levels^16–18^, and possibly higher AGE accumulation rate^19^ than controls. A recent longitudinal cohort study showed that AGEs can predict psychotic symptom trajectories among drug-naïve adolescents^20^. Thus, there is a need to understand the role of AGEs in the association between muscle strength and psychosis to elucidate the biological mechanisms and early modifiable factors of psychosis.

In this study, we first examined the direction of relationship between muscular underdevelopment and AGE accumulation among adolescents (Study 1). Then, we investigated the role of AGEs in the longitudinal association between muscular strength and psychotic symptoms among adolescents (Study 2). To the best of our knowledge, this is the first study to examine the role of AGEs in the longitudinal association between muscular strength and the development of psychotic phenomena among adolescents. We hypothesized that AGEs mediate the relationship between lower muscular strength and the development of psychosis.

## Results

For Study 1, we examined the direction of relationship between handgrip strength and urinary pentosidine levels, as a representative AGEs, to determine the temporal order of these factors using the data from 1542 participants. Table 1 shows the characteristics of participants who had no missing data on baseline handgrip strength and urinary pentosidine levels. Information of the participants who were included in and excluded from the analysis were summarized in supplementary information.

**Table 1:** Characteristics of participants in Study 1.

Variables	*N*	
Grip strength at age 12 (kg, mean ± SD)^a^	1542	18.7 ± 4.3
Grip strength at age 14 (kg, mean ± SD)^a^	1251	26.3 ± 6.8
Pentosidine in urine at age 12 (pmol/mg・Cr, mean ± SD)^b^	1542	5.9 ± 1.8
Pentosidine in urine at age 14 (pmol/mg・Cr, mean ± SD)^b^	1094	5.0 ± 1.5
Body mass index at age 12	1542	17.8 ± 2.4
Sex (male/female, N)	1542	861/681

^a^Handgrip strength was measured using the digital handgrip meter with the most powerful posture. We calculated the average value for both hands.

^b^Urinary pentosidine levels were determined by a high-performance liquid chromatography assay.

*SD* standard deviation.

**Fig. 1:**
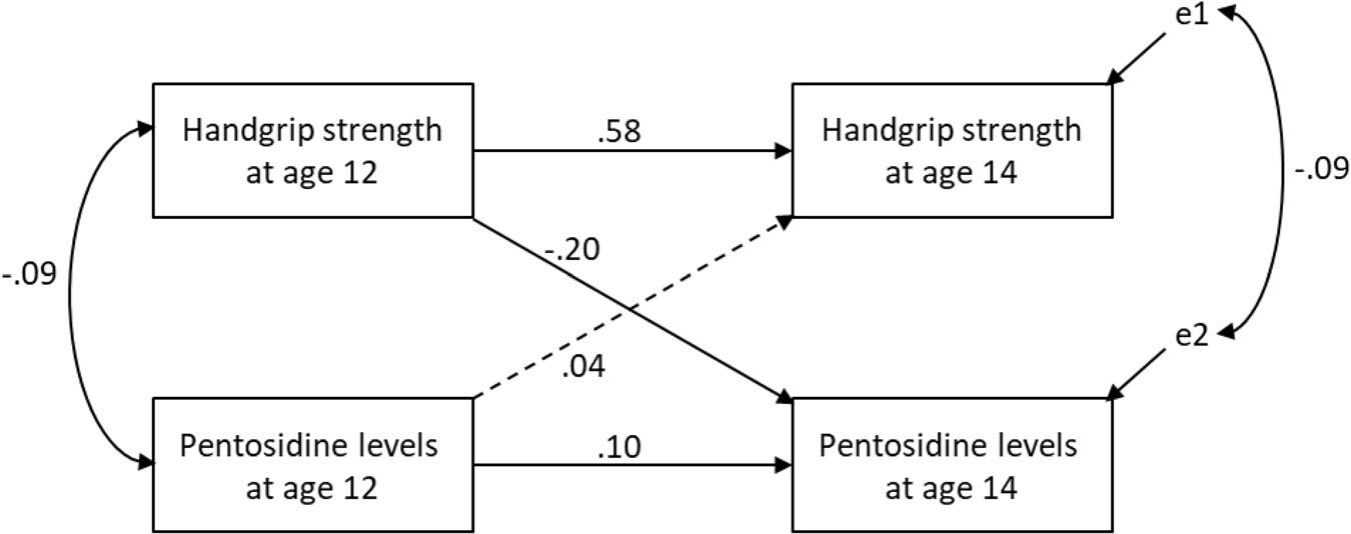
Cross-lagged panel model showing the direction of association between handgrip strength and pentosidine levels. Note: Solid black line, path coefficient is statistically significant (*p* < 0.05); dotted line, path coefficient is not significant (*p* ≥ 0.05).

Figure 1 shows the path diagram describing the saturated cross-lagged model (CFI = 1.00), which consists of handgrip strength and urinary pentosidine levels at ages 12 and 14. Handgrip strength at age 12 negatively predicted urinary pentosidine levels at age 14 (β = −0.20, *p* < 0.001), whereas urinary pentosidine levels at age 12 did not significantly predict handgrip strength at age 14 (β = 0.04, *p* = 0.062). The autoregressive effects of handgrip strength and pentosidine levels from ages 12 to 14 were significant (β = 0.58, *p* < 0.001; β = 0.10, *p* < 0.001, respectively).

Next, adjustment for the effect of body mass index (BMI) on the result of the cross-lagged model was performed because handgrip strength was significantly correlated with BMI at age 12 (correlation coefficient: r = 0.34, *p* < 0.001). Handgrip strength at age 14 was strongly correlated with body muscle mass at age 14 (mean = 38.4 ± 5.8 kg; correlation coefficient: r = 0.70, *p* < 0.001), which imply that handgrip strength could be an indicator of muscle strength. Regression analysis showed that handgrip strength at age 12 negatively predicted urinary pentosidine levels at age 14 (β = −0.19, *p* < 0.001), even after adjustment for BMI at age 12 that did not predict urinary pentosidine levels at age 14 (β = −0.05, *p* = 0.149).

Table 2 shows the characteristics of the participants with no missing data for the main predictor and investigated outcome variables for Study 2. The participants who were included in and excluded from the analysis showed no significant differences in sex, handgrip strength, or TP scores at age 12 (*p* > 0.05). The TP scores gradually increased with age (repeated measures of one-way analysis of variance: F[1.858, 22.443] = 19.196, *p* < 0.001). Urinary pentosidine levels decreased from ages 12 to 13 years (paired *t*-test: t[198] = 10.855, *p* < 0.001).

**Table 2:** Characteristics of participants in Study 2.

Variables	*N*	
Handgrip strength at age 12 (kg, mean ± SD)^a^	256	18.7 ± 4.2
Thought problems score at age 12 (mean ± SD)^b^	250	0.8 ± 1.5
Thought problems score at age 13 (mean ± SD)^b^	253	1.0 ± 1.7
Thought problems score at age 14 (mean ± SD)^b^	256	1.4 ± 1.7
Urinary pentosidine level at age 12 (pmol/mg・Cr, mean ± SD)^c^	206	6.1 ± 1.8
Urinary pentosidine level at age 13 (pmol/mg・Cr, mean ± SD)^c^	247	4.4 ± 1.7
Sex (male/female, N)	256	143/113

^a^Handgrip strength was measured at 12 years of age by using the digital handgrip meter with the most powerful posture. We calculated the average value for both hands.

^b^Thought problems were measured using the subscale of the child behavior checklist (CBCL) completed by the parents.

^c^Urinary pentosidine levels were determined by a high-performance liquid chromatography assay.

*SD* standard deviation.

**Fig. 2:**
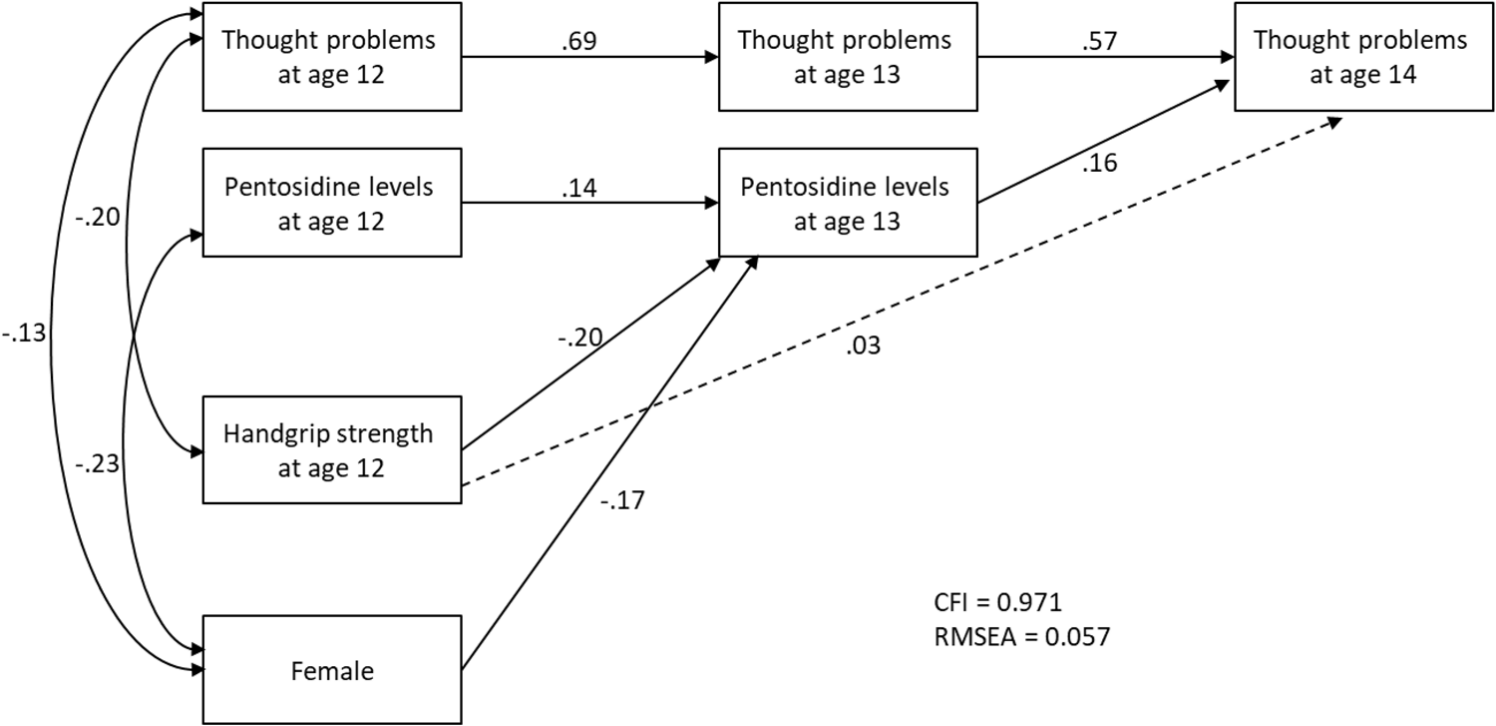
Path diagram describing the indirect effect between handgrip strength and thought problems via pentosidine. Note: solid black line, path coefficient is statistically significant (*p* < 0.05); dotted line, path coefficient is not significant (*p* ≥ 0.05). CFI: comparative fit index, RMSEA: root mean square error of approximation.

The details of the correlation coefficients among all variables in a saturated path model and the results of the analysis without adjusting for covariates are summarized in Table S1 and supplementary information, respectively. Female sex was negatively associated with TP scores at ages 12 and 14, and with urinary pentosidine levels at ages 12 and 13. Figure 2 shows the path diagram describing the mediating effect of urinary pentosidine levels after adjusting for covariates. This model demonstrated a good or reasonable fit (CFI = 0.971, RMSEA = 0.057). Handgrip strength at age 12 negatively predicted urinary pentosidine levels at age 13 (β = −0.20, *p* = 0.001), and urinary pentosidine levels at age 13 positively predicted TP scores at age 14 (β = 0.16, *p* = 0.002), even after adjusting for sex differences and baseline levels of urinary pentosidine and TP scores. The indirect effect between handgrip strength at age 12 and TP at age 14 via urinary pentosidine levels was statistically significant (standardized indirect effect = −0.032, *p* = 0.025), while direct effect was not significant (β = 0.033, *p* = 0.520). Supplemental Fig. S1 shows the scatter plots for the main hypotheses, indicating the longitudinal association between handgrip strength at age 12 and urinary pentosidine levels at age 13 (correlation coefficient: *r* = 0.20, *p* < 0.01), and between urinary pentosidine levels at age 13 and thought problems score at age 14 (correlation coefficient: *r* = 0.25, *p* < 0.01).

## Discussion

Using prospective cohort data from a general adolescent population, Study 1 showed that low muscular strength could predict an increase in AGEs levels while BMI could not. In Study 2, we found an indirect effect via AGEs between handgrip strength and TP among adolescents. Furthermore, our findings support the hypothesized path, where low muscle strength is associated with increased AGE levels, which then results in psychosis.

Puberty is a critical period of significant muscle development, and underdevelopment during this period can affect the brain and body. Taken together, our findings highlight the importance of the muscle-brain axis, which is further supported by a recent review summarizing the effect of exercise in protecting mental health in youth at risk for psychosis^21^. Additionally, another review indicated that muscle-induced peripheral factors enable direct crosstalk between muscle and brain function^22^. These evidence provide new biological insights into Kretchmer’s body-type theory and explain one mechanism by which muscle underdevelopment causes psychotic phenomena.

Previous cross-sectional studies have demonstrated a relationship between AGEs and low muscle strength^13,14^, although its direction is unclear. This is the first study to suggest a longitudinal relationship between handgrip strength and AGEs. The skeletal muscle is the largest metabolic organ and accounts for 30% of the basal metabolic rate^15^. Moreover, it can import and store glucose as glycogen in an insulin-dependent manner. Therefore, low skeletal muscle strength, indicated by low handgrip strength, could increase blood glucose levels and lead to AGE production, as previously reported^23^. Furthermore, the skeletal muscle acts as a storage tissue for vitamin B6. In mammals, 70%–80% of vitamin B6 in the body is stored in the skeletal muscles^24^. Vitamin B6 also inhibits AGE formation by blocking the oxidative degradation of the Amadori intermediate of the Maillard reaction^25^. Therefore, low skeletal muscle strength could induce AGE accumulation due to vitamin B6 deficiency. Interestingly, glucose metabolism disturbances and vitamin B6 reduction have been reported in patients with schizophrenia^26,27^.

Previous studies have reported AGE accumulation in patients with schizophrenia^16–18^, and higher AGE accumulation rate^19^. Furthermore, we recently reported that increased AGE levels predict persistent psychotic symptoms in drug-naïve adolescents^20^. The role of AGEs in the development of psychosis may involve brain inflammation. During adolescence, inflammatory system enhancement via the receptor for AGEs (RAGE) system can significantly damage brain development and cause psychosis. RAGE is a single transmembrane receptor that is expressed in skeletal muscle cells, neurons, and endothelial cells^28,29^. Additionally, AGEs binding to RAGE enhances intracellular oxidative stress, which in turn activates neuroinflammatory molecules, including the transcription factor NF-κB, via the RAS/MAP kinase pathway^30^. Furthermore, patients with schizophrenia have been reported to show decreased plasma levels of soluble RAGE, which inhibits inflammation due to the lack of a membrane-bound domain^31^. These findings suggest that the AGEs–RAGE axis could be associated with the development of psychotic symptoms. Moreover, AGEs are known to be associated with various age-related diseases through inflammation and vascular damage, and elevated AGEs levels may provide a mechanism to explain the association between psychosis risk and cardiovascular disease^18^.

The observed longitudinal relationship between muscle weakness and psychotic symptoms mediated by AGEs suggests the possibility of interventions to prevent psychosis onset. Handgrip strength is a simple and non-invasive measure of skeletal muscle strength. Therefore, it can be applied in community settings, including schools and homes. Although the usefulness of handgrip strength as a screening tool for psychotic symptoms has not yet been investigated, it could allow for early identification of high-risk adolescents and prevention of subsequent psychotic symptoms. Given the inverse relationship between moderate exercise and AGEs^32^, moderate exercise during adolescence could be effective intervention for AGE reduction, which could subsequently prevent the occurrence of psychotic symptoms. Furthermore, daily intake of vitamin B6, an AGE inhibitor, could also be effective in preventing psychosis and has shown clinical improvement in schizophrenia patients with high plasma AGE levels^33^. Taken together, high-risk adolescents presenting with low handgrip strength could benefit from preventive interventions targeting AGEs. There is a need for further intervention studies targeting AGEs or muscular strength among adolescents.

### Strengths and limitations

The strengths of this study are mainly attributed to its longitudinal design and population-based prospective cohort data. This allowed us to determine the relationships among muscular strength, psychotic symptoms, and AGEs. Other strengths include the use of handgrip strength, which allows for an easy and precise measurement that reflects the total body skeletal muscle mass. Moreover, it is especially feasible to assess the adolescent population in the community. However, this study had several limitations. First, the only AGE that was measured was pentosidine. Elevated pentosidine has been repeatedly confirmed in patients with schizophrenia and is considered to be the most robust AGE marker^16,17^. Second, approximately 19%, and 29% of the data on handgrip strength and urinary sample, respectively, were missing. Third, the assessment of TP was not performed through clinical interviews by professionals but rather through a caregiver’s ratings with the CBCL. Although CBCL was a standardized, widely-used measure, parents may underestimate their child’s TP and it has not been validated within this study. Fourth, we adjusted the TP at age 12 years in the path analysis, however, this could not completely eliminate the effects of preceded psychotic symptoms, which may exist before the initiation of the present study, on the results of this study. Similarly, the path analysis was not adjusted for the environmental factors including smoking or substance use. Although the indirect effect between handgrip muscle strength and TP via glycation stress was found in the present study, further experimental studies would be needed to confirm the mediation pathways and the role of each factor in the etiology of psychotic symptoms.

## Conclusions

In conclusion, our study provides evidence that adolescents with low muscular strength are at a risk of developing psychotic symptoms, which could be mediated by AGEs. Future studies need to examine whether interventions focused on muscular strength prevent the accumulation of AGEs and prevent the development of psychosis.

## Methods

### Study 1

#### Study design, participants, and survey procedure

This study used data from a prospective population-based birth cohort study (Tokyo TEEN Cohort: TTC)^34^ (URL: http://ttcp.umin.jp) of youth living in three municipalities in metropolitan Tokyo (Setagaya, Mitaka, and Chofu). The TTC is a multidisciplinary survey of adolescents, ages 10, 12, and 14, aimed at investigating adolescent health and development. We randomly sampled 3,171 households with 10-year-old early adolescents using the basic resident register in the three municipalities; eligible residents were adolescents born between September 2002 and August 2004. A survey of youth at age 10 was conducted between October 2012 and January 2015. When these adolescents were 12 and 14 years of age, 3,007 households participated in the second wave of the study (follow-up rate: 94.8%), and 2,667 households participated in the third wave of the study (follow-up rate: 84.1%), respectively. We sent letters of invitation to participants around their birthday of the target age at each wave. A trained interviewer then visited their homes, and the survey was completed over two visits in each wave. The TTC is based on three research institutes: the Tokyo Metropolitan Institute of Medical Science, The University of Tokyo, and SOKENDAI (The Graduate University for Advanced Studies). This survey was approved by the ethics committees of all three institutes.

#### Measurement of handgrip strength at ages 12 and 14 years

Handgrip strength is indicative of skeletal muscle strength^10^. Data regarding handgrip strength were collected in the whole-sample survey of the TTC through home visits when the participants were 12 and 14 years old. Handgrip strength was measured using a digital handgrip meter (MCZ-5041; Macros, Tokyo, Japan) with the most powerful posture. Trained examiners performed measurements using a standard procedure^35^. The average handgrip strength of both hands was calculated. To confirm the concurrent validity of handgrip strength for evaluating muscle strength, we examined the correlation between handgrip strength and body muscle mass, which was measured using a body composition monitor (BC-759; TANITA, Tokyo, Japan).

#### Measurement of urinary pentosidine levels at ages 12 and 14 years

During the surveys conducted at ages 12 and 14, participants’ first urine in the morning was collected and immediately frozen at −80 °C. AGE levels were evaluated by measuring urinary pentosidine levels. In brief, 250 μL of the sample was hydrolyzed in 250 μL of 12 N hydrochloric acid, purified by solid-phase extraction, and then dried with nitrogen flow, followed by dissolution in 500 μL of 1% heptafluorobutyric acid. A sample was injected into a high-performance liquid chromatography system (ACQUITY UPLC H-Class, Waters Inc.) and fractionated on a C18 reverse-phase column. The effluent was monitored at excitation and emission wavelengths of 335/385 nm by using a fluorescence detector. Commercially available pentosidine (Cayman Chemical) was used to obtain a calibration curve. Pentosidine levels were corrected for renal function using urine creatinine levels.

#### Body mass index at age 12 years

Body mass index (BMI) is an indicator of body shape reflecting fat mass and could be a potential confounding factor in the relationship between handgrip strength and AGE levels in adolescence. BMI is associated with not only fat mass, but also fat-free mass during adolescence^36^. A positive association has been suggested between BMI and hand grip strength in adolescence^37^. Previous studies have also suggested that BMI is negatively associated with AGE levels^38^. To clarify the effect of muscle strength on AGEs, we set BMI as a covariate.

#### Statistical analysis

Cross-lagged path analysis was used to infer the direction of associations between handgrip strength and urine pentosidine levels from longitudinal data. Cross-lagged models can analyze reciprocal relationships between two or more observed variables measured at two or more distinct time points, including both autoregressive effects and cross-lagged effects within the model^39^. In the present study, we set a path model to examine the cross-lagged effects of handgrip strength and urine pentosidine levels. The path model included the effect of handgrip strength at ages 12 to 14 (autoregressive effect of handgrip strength), urine pentosidine levels at ages 12 to 14 (autoregressive effect of urine pentosidine level), handgrip strength at age 12 to urine pentosidine level at age 14 (cross-lagged effect of handgrip strength on urine pentosidine level), and urine pentosidine level at age 12 to handgrip strength at age 14 (cross-lagged effect of urine pentosidine level on handgrip strength). Regression analysis was conducted to confirm the effect of muscle strength on AGE levels by using BMI as a covariate. The regression model included handgrip strength at age 12 as a predictor and urine pentosidine level at age 14 as the outcome. The model also included BMI at age 12 as a covariate. The correlation between handgrip strength and BMI at age 12 was set in the regression model. We adopted a full-information maximum likelihood estimation procedure to handle missing data under the assumption of missing data at random^40^. Statistical analyses were performed using IBM SPSS Statistics for Windows (version 26.0) and Amos 24.0 (IBM Corp., Armonk, New York, USA). A two-tailed test was performed with a significance level (α) set at 0.05.

### Study 2

#### Study design, participants, and survey procedure

This study was conducted as part of the population-based biomarker subsample study of the TTC study (http://ttcp.umin.jp) (pb-TTC), which included 345 adolescents (mean age ± SD = 13.5 ± 0.6 years) and their caregivers (mainly mothers). This study examined biological markers, including AGE levels, in the adolescents’ urine, and their main caregivers rated their TP as a psychotic risk indicator.

Participants in the pb-TTC were enrolled from a larger sample of the TTC project, which was a large-scale population-based birth cohort study conducted in the Tokyo metropolitan area using 3,171 adolescent–caregiver dyads^34,41,42^. There were no significant differences in age, sex, or socioeconomic status (*p* > 0.05) between the pb-TTC subsample and the TTC population. We longitudinally followed adolescents aged 13 and 14 years in the pb-TTC subsample. Among the 345 individuals who participated in the survey at 13 years, 282 (82%) completed the survey at 14 years of age.

We obtained written informed assent and consent from each participant and their main caregiver, respectively, before study participation. The authors assert that all procedures contributing to this work comply with the ethical standards of the relevant national and institutional committees on human experimentation and with the Helsinki Declaration of 1975, as revised in 2008. All procedures involving human subjects/patients were approved by the Research Ethics Committee of the Tokyo Metropolitan Institute of Medical Science.

#### Main predictor: Handgrip strength at age 12 years

Handgrip strength measurements were performed in the same manner as in Study 1.

#### Outcome: Thought problems at age 14 years

As the main outcome variable, TP at 14 years of age were measured using seven items in the CBCL completed by the main caregiver^43,44^. The scores for TP of the CBCL can be used to screen for early detection of the risk of developing psychotic symptoms in youth^45^. The TP subscale includes the following questions: (i) “Can’t get his/her mind off certain thoughts; obsessions”, (ii) “Hears sounds or voices that aren’t there”, (iii) “Repeats certain acts over and over; compulsions,” (iv) “Sees things that aren’t there”, (v) “Stares blankly,” (vi) “Strange behavior” and (vii) “Strange ideas.” All responses were rated on a 3-point scale: not true = 0, somewhat/sometimes true = 1, and very true/often true = 2. The TP score was defined as the total score of the seven items (possible range: 0–14).

#### Mediator: Urinary pentosidine level at age 13 years

During the survey, at 13 years of age, the participants’ first urine in the morning was collected and immediately frozen at −80 °C. Pentosidine measurements were performed in the same manner as for adolescents aged 12 and 14 years.

#### Covariates

We adjusted for sex as a potential confounding variable of the associations among handgrip strength, urinary pentosidine levels, and TP in adolescents. Moreover, we included TP scores at the age of 12 and 13 years as well as urinary pentosidine levels at age 12 years as covariates to adjust for the autoregressive effect on each factor. Urinary pentosidine levels and TP scores at 12 years of age were measured in the whole-sample survey. Furthermore, the TP scores at 13 years of age were measured in the subsample survey.

#### Statistical analysis

Path analyses were conducted to examine the mediating effect of urinary pentosidine levels on the association between handgrip strength and TP. Model fit was assessed using two common indices: the comparative fit index (CFI) and the root mean square error of approximation (RMSEA). A CFI value > 0.9 is indicative of good model fit, and RMSEA values <0.05 and <0.10 indicate good and reasonable model fit, respectively. Potential mediating effects were assessed by calculating the indirect mediator effect on the relationship between handgrip strength and TP. We calculated the indirect effect by multiplying the path coefficient between the handgrip strength and urinary pentosidine levels and between the urinary pentosidine levels and TP scores. The Sobel test was performed to test the mediation effect of urinary pentosidine levels^46–48^. Mediation effects were tested with and without adjustment for covariates.

## Supplementary information

Supplemental Table S1

Supplemental Figure S1

Supplemental information

## Data Availability

The data that support the findings of this study are available from the corresponding author, A.N., upon reasonable request.
