# Supplementary material for: Role of advanced glycation end products in the longitudinal association between muscular strength and psychotic symptoms among adolescents
Source: Schizophrenia (Heidelb). 2022 Apr 27;8(1):44. doi: 10.1038/s41537-022-00249-5 (PMC9261085; doi:10.1038/s41537-022-00249-5)
Supplement: Supplementary file 1 — Supplemental Table S1 [file 41537_2022_249_MOESM1_ESM.docx]

| **Table S1. Correlation coefficients among all variables** | | | | | | | | | | | | | |  |
| --- | --- | --- | --- | --- | --- | --- | --- | --- | --- | --- | --- | --- | --- | --- |
|  | 1 | 2 | | 3 | | 4 | | 5 | | 6 | | 7 | | |
| 1: Handgrip strength at age 12 | 1 | -0.191 | ** | -0.093 |  | -0.051 |  | -0.084 |  | -0.200 | ** | -0.060 |  | |
| 2: Thought problems score at age 12 |  | 1 |  | 0.687 | ** | 0.494 | ** | 0.086 |  | 0.218 | ** | -0.135 | * | |
| 3: Thought problems score at age 13 |  |  |  | 1 |  | 0.585 | ** | 0.065 |  | 0.184 | ** | -0.066 |  | |
| 4: Thought problems score at age 14 |  |  |  |  |  | 1 |  | 0.064 |  | 0.253 | ** | -0.155 | * | |
| 5: Urinary pentosidine level at age 12 |  |  |  |  |  |  |  | 1 |  | 0.204 | ** | -0.246 | ** | |
| 6: Urinary pentosidine level at age 13 |  |  |  |  |  |  |  |  |  | 1 |  | -0.192 | ** | |
| 7: Females |  |  |  |  |  |  |  |  |  |  |  | 1 |  | |
| ** p < 0.01, * p < 0.05  Correlation coefficients were estimated using a full information maximum likelihood (FIML) estimation procedure to handle missing data under the assumption of missing at random (MAR). | | | | | | | | | | | | | | |
