# Supplementary material for: Role of advanced glycation end products in the longitudinal association between muscular strength and psychotic symptoms among adolescents
Source: Schizophrenia (Heidelb). 2022 Apr 27;8(1):44. doi: 10.1038/s41537-022-00249-5 (PMC9261085; doi:10.1038/s41537-022-00249-5)
Supplement: Supplementary file 2 — Supplemental Figure S1 [file 41537_2022_249_MOESM2_ESM.pdf]

Supplemental Figure S1. Longitudinal associations among handgrip strength, urinary pentosidine levels and thought problems.

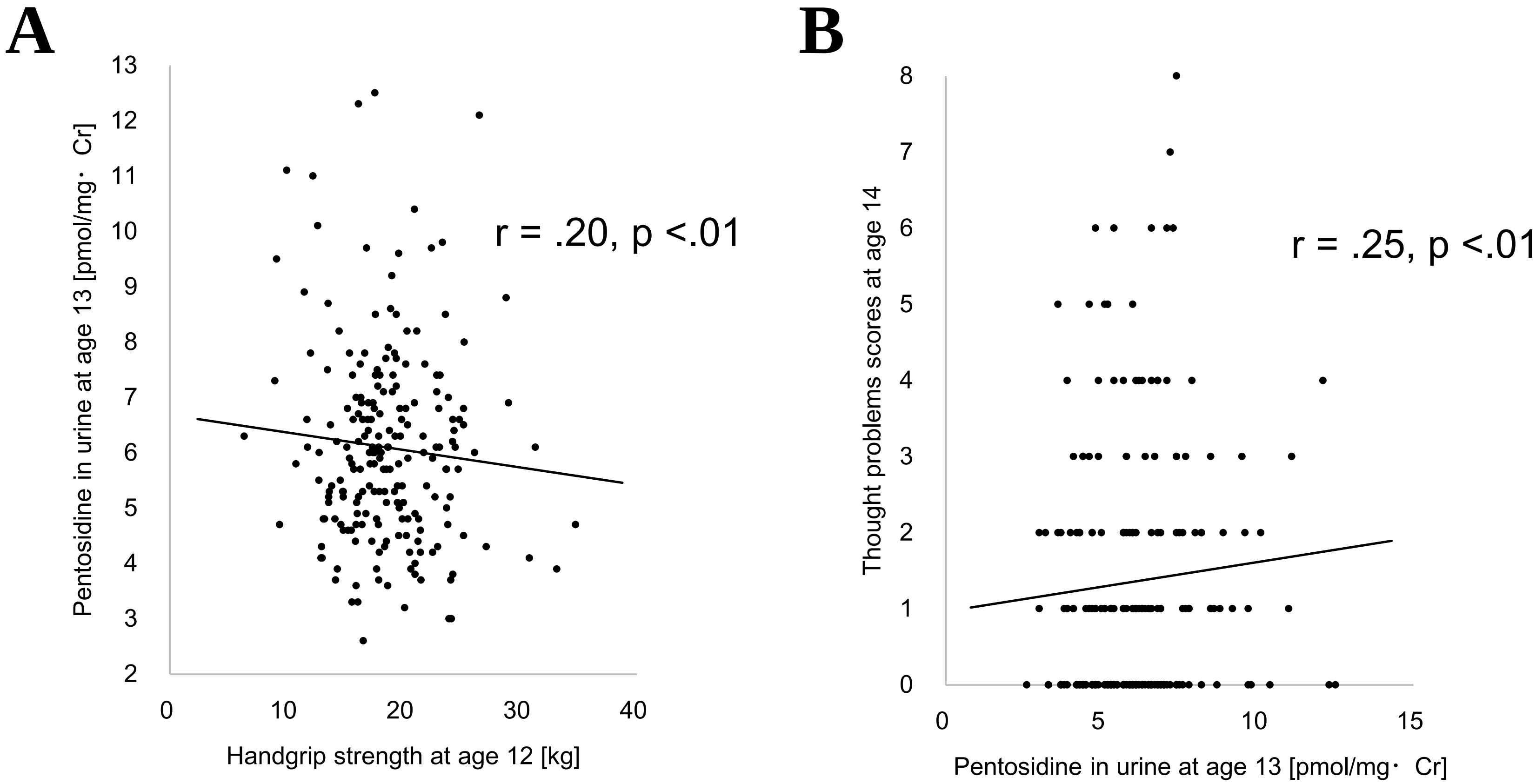

Supplemental Figure S1.

The scatter plots show the longitudinal association between handgrip strength at age 12 and urinary pentosidine levels at ages 13 (A), and between urinary pentosidine levels at age 13 and thought problems score at age 14 (B).
