# Supplementary material for: Role of advanced glycation end products in the longitudinal association between muscular strength and psychotic symptoms among adolescents
Source: Schizophrenia (Heidelb). 2022 Apr 27;8(1):44. doi: 10.1038/s41537-022-00249-5 (PMC9261085; doi:10.1038/s41537-022-00249-5)
Supplement: Supplementary file 3 — Supplemental information [file 41537_2022_249_MOESM3_ESM.docx]

**Supplemental information**

1. Information of the participants who were included in and excluded from the analysis in study 1.

Participants who were included in and excluded from the analysis in study 1 showed no significant differences in handgrip strength or urinary pentosidine levels at ages 12 and 14 (p > 0.05), although there were more male participants in the included group than in the excluded group (56% vs. 50%; χ^2^ = 8.99, p = 0.003).

1. The results of the path analysis without adjusting for covariates in study 2.

In a saturated path model including handgrip strength at age 12, urinary pentosidine levels at age 13, and TP scores at age 14 (without adjusting for covariates), the mediation effect of urinary pentosidine levels on the relationship between handgrip strength and TP scores was statistically significant (standardized indirect effect = -0.051, p = 0.012), while a direct path between handgrip strength and TP scores was not (β = 0.000, p = 0.997).
